# Sequential Topology Optimization and Generative Design with Finite Element Validation for Lightweight PA6 Roller Conveyor Components

**DOI:** 10.3390/ma19163401

**Published:** 2026-08-11

**Authors:** Ziad Alamin Emhmed Alhashmi, Aleksandra Mitrovic, Marko Tasic, Zorana Golubovic, Nenad Mitrovic

**Affiliations:** 1 Faculty for Information technologies and Engineering, University Union—Nikola Tesla, 11000 Belgrade, Serbia; eng.zaid.alhashmi@gmail.com; 2 Department of Forensic Engineering, Faculty of Forensic Sciences and Engineering, CBRNE Center of Excellence, University of Criminal Investigation and Police Studies, 11000 Belgrade, Serbia; aleksandra.mitrovic@kpu.edu.rs; 3 The Academy of Applied Studies Polytechnic, 11000 Belgrade, Serbia; mtasic@politehnika.edu.rs; 4 University of Belgrade, Faculty of Mechanical Engineering, 11000 Belgrade, Serbia; zzgolubovic@mas.bg.ac.rs

**Keywords:** topology optimization, generative design, polyamide-6 (PA6), finite element analysis, lightweight structures, conveyor idler roller

## Abstract

**Highlights:**

- Sequential topology optimization and generative design reduced PA6 roller mass by 37% (1345 → 848 g).
- Generative design reversed the topology optimization stiffness loss, cutting displacement by 35%.
- The optimized roller achieved a safety factor of ~3.7 against yield under service loading.
- Topology optimization alone increased displacement from 3.4 to 4.4 mm, showing its limits.
- A reproducible FEA-validated workflow links mass reduction to retained structural performance.
- Pairing topology optimization with generative design avoids stiffness loss risked by topology optimization alone.
- Lighter PA6 rollers could reduce energy demand across large-scale mining conveyor systems.

**Abstract:**

Conveyor idler rollers are conventionally manufactured from steel, contributing substantially to the overall mass, energy consumption, and operating cost of belt conveyor systems. A sequential optimization methodology combining topology optimization and generative design is presented to reduce the mass of a polyamide-6 (PA6) idler roller while preserving its structural performance under representative service loading. Starting from a conventional baseline model (1345 g), topology optimization identified low-utilization regions for material removal but introduced an intermediate stiffness penalty, increasing maximum displacement from 3.4 mm to 4.4 mm. Generative design subsequently reorganized the remaining material along the principal load paths, yielding a final component mass of 848 g, a 37% reduction relative to the baseline, while simultaneously reducing maximum displacement to 2.2 mm, a 35% improvement over the original design. The optimized component sustained a maximum von Mises stress of 16.3 MPa under the applied load, corresponding to a safety factor of approximately 3.7 relative to the material’s yield strength. These results, consistent with mass and stiffness improvements reported for generative design applied to comparable load-bearing components, demonstrate that sequential topology optimization and generative design can recover and exceed the stiffness of a conventional design while substantially reducing mass, offering a reproducible pathway toward lighter, more energy-efficient polymer-based conveyor components.

## 1. Introduction

Belt conveyor systems are essential for bulk material transport in mining and industrial applications, where continuous operation, reliability, and energy efficiency are critical. Idler rollers support the belt and transmitted loads, but conventional steel designs increase inertia, noise, and energy consumption. Polymer rollers have been proposed as a lighter alternative, reducing wear and improving efficiency while maintaining structural integrity and durability [1,2,3,4,5]. Computational approaches such as generative design and topology optimization enable material redistribution along load paths, facilitating lightweight solutions beyond conventional design limits [6,7,8]. Additionally, polymer materials offer low density and vibration damping, mitigating noise and dynamic loading while ensuring adequate mechanical performance [9,10,11]. The integration of optimized geometric design with advanced polymer materials thus represents a promising pathway toward more efficient and durable conveyor roller systems.

Despite these advantages, existing studies on polymer-based idler rollers have also identified several persistent limitations that constrain their broader industrial adoption. Polymer idler roller bodies remain susceptible to abrasion, fatigue, and microstructural degradation under extreme temperature conditions, with material wear directly linked to increased friction and belt damage in severe cases [12]. PA6 specifically exhibits high moisture sensitivity, which can alter its mechanical properties relative to as-supplied datasheet conditions. Furthermore, idler components represent a substantial proportion of overall conveyor system cost, motivating continued research into optimization approaches that jointly consider structural performance, service life, and economic factors [13].

Conventional manufacturing approaches supported by Computer-Aided Design (CAD) and Computer-Aided Manufacturing (CAM) systems have traditionally guided the development of conveyor components; however, these methods impose geometric constraints that limit further weight reduction and structural optimization [3,4,14]. Recent advances in digital manufacturing enable the direct production of complex, customized geometries from digital models, expanding feasible engineering solutions [15,16]. Optimization-driven design approaches have achieved significant mass reduction while maintaining structural functionality, reducing both material consumption and manufacturing waste [17,18]. Generative design (GD) methods, in particular, allow systematic exploration of design spaces through iterative parameter modification and performance evaluation [19,20,21,22]. Accurate prediction of optimized configurations requires continuous refinement of modeling parameters and validation through established numerical techniques [23].

Structural evaluation of optimized geometries is commonly performed using analytical formulations and numerical methods, most notably the Finite Element Analysis (FEA), which enables computational assessment of stresses, deformations, and overall structural response [24,25]. Analytical approaches, such as classical beam theory and stress–strain formulations, offer valuable insight into the structural dynamics, load distribution, and stress analysis of conveyor rollers. However, these methods are limited in capturing nonlinear effects, contact interactions, and complex material behaviors, especially in polymer and composite systems. To address these limitations, FEA has been used to simulate the mechanics of conveyor components, allowing detailed evaluation of stresses, deformations, and load transfer under operational conditions [26].

While the combined application of topology optimization and generative design has been demonstrated on metallic components [21], their sequential application to a polymer, load-bearing rotating component under representative industrial service loading has not yet been reported.

The aim of this research is to reduce the mass of a PA6-based conveyor idler roller through sequential application of topology optimization and generative design, while preserving structural stiffness and load-bearing capacity, with finite element analysis used to quantify and validate the resulting mechanical performance across the baseline, topology-optimized, and generative design stages.

## 2. Materials and Methods

**Figure 1:**
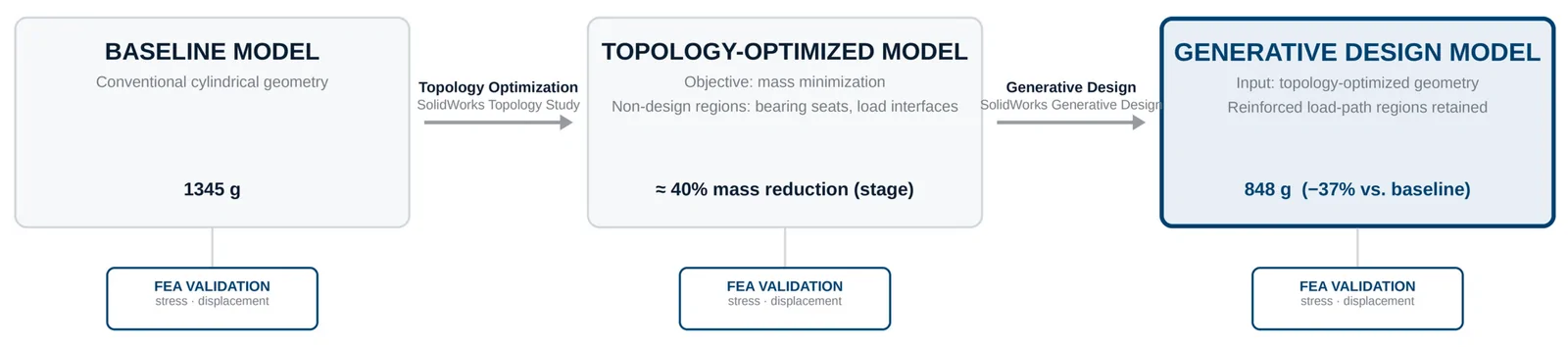
Overview of the sequential optimization workflow: the conventional baseline model was processed through topology optimization (objective: mass minimization) to generate an intermediate geometry, which was subsequently used as the input for generative design to produce the final optimized model. Finite element analysis (FEA) was performed independently at each stage to validate stress and displacement performance.

In this study, a structured research methodology was used to develop and evaluate an optimized PA6 conveyor roller component. The approach integrates sequential and interrelated stages, including defining operational loading and boundary conditions, selecting materials and creating baseline models, applying topology optimization and generative design techniques, and numerically validating results through finite element analysis. Where applicable, numerical findings were compared with the available experimental or literature data to assess structural performance under representative service conditions. For clarity, the structural evaluation is presented in three sequential stages, the conventional (baseline) model (Phase A), the topology-optimized model (Phase B), and the generative design model (Phase C), allowing direct comparison of mass, stress, and displacement across the optimization process (Figure 1).

### 2.1. Component Definition and Modeling Interfaces

The analyzed component in this research is a supporting idler roller used in belt conveyor systems operating in surface mining environments. In these systems, rollers provide continuous support to the conveyor belt and the transported bulk material along the conveyor route, while rotation occurs around a stationary shaft supported by rolling bearings at both ends.

The roller assembly consists of a cylindrical shell, a fixed shaft, and bearing units positioned at the end interfaces connecting the component to the conveyor supporting structure. During operation, the shaft remains stationary relative to the structure, while the roller shell rotates under belt motion.

In the numerical model, the bearing locations were represented as support interfaces transmitting reaction forces to the conveyor structure. These supports model bearing constraints rather than rigid clamped conditions, reflecting the actual installation of rollers within suspended or fixed garland assemblies. Similar finite element modeling strategies have been applied in the literature to conveyor components, including large-scale belt conveyor drums, where accurate representation of support interfaces and operational loading is critical for reliable stress and deformation analysis [27].

Operational loading of conveyor rollers is governed primarily by forces acting in the vertical plane, originating from the combined weight of the conveyor belt, transported material, and belt tension effects. Standard analytical design procedures for conveyor rollers treat the dominant load component as radial, while axial loading is generally neglected and considered secondary, arising mainly from belt misalignment or operational deviations.

Consequently, the loading applied in the finite element analysis represents an equivalent radial operational load transmitted from the conveyor belt to the roller surface and further transferred through the bearings to the supporting structure. The shaft ends were modeled as pin (hinge) supports, consistent with the bearing-type connection to the conveyor structure. A preliminary analytical calculation was performed in accordance with the CEMA standard methodology for troughed belt conveyors, using representative parameters for a heavy-duty surface-mining application (belt tension of 19,200 N, a transported-material loading of 245.1 kg/m, and a troughing angle of 30°). Under these conditions, the center (horizontal) roller of the troughing idler set, which carries the largest share (70%) of the total radial load, was found to be subjected to a radial force of approximately 6146 N. The load applied in the finite element analysis (2000 N applied to the quarter-symmetry model, corresponding to a full bearing reaction of 8000 N) was adopted as a representative, conservative service value for the structural evaluation of the roller investigated in this study, exceeding the total calculated radial force obtained from this preliminary analytical calculation.

To justify the use of static structural analysis rather than a dynamic approach, a modal analysis of the roller assembly was additionally performed. The operational excitation frequency, corresponding to a nominal rotational speed of 600 rpm (10 Hz), was compared with the first four natural frequencies of the structure, obtained as 164.85 Hz, 183.68 Hz, 248.55 Hz, and 368.24 Hz, respectively. Since the excitation frequency lies far below the lowest natural frequency, no resonance-driven amplification of displacement or stress is expected under normal operating conditions, and the structural response can reasonably be treated as quasi-static for the purposes of this study.

### 2.2. Material Selection

Conveyor rollers (idlers) are essential components of belt conveyor systems, providing continuous support to the conveyor belt and transported material and significantly influencing operational performance and reliability [28,29]. To meet the need for lightweight and mechanically reliable conveyor components, polymer materials were considered as alternatives to conventional metal solutions. Polyamide-6 (PA6), a high-strength thermoplastic engineering polymer, was selected for its favorable combination of strength, wear resistance, and suitability for load-bearing applications in conveyor systems (Table 1). PA6 was selected among candidate engineering polymers primarily on the basis of its established mechanical properties and its wide commercial availability, both of which support its practical suitability for load-bearing conveyor components. The mechanical properties listed in Table 1 were obtained from the manufacturer’s material datasheet and were adopted as input parameters for the subsequent numerical analysis in this study.

### 2.3. Baseline Model

The baseline geometry was modeled as a conventional cylindrical roller manufactured using standard CNC design principles. The component consists of a uniform cylindrical body with stepped diameters at both ends, forming shoulder regions for bearing interfaces, while the central section serves as the primary load-bearing span.

A representative operational load of 2000 N per bearing was applied to evaluate the structural response. The model was defined with rotational symmetry about the longitudinal axis of the roller. Boundary conditions were imposed at the end interfaces to represent constrained support regions corresponding to bearing locations.

### 2.4. Application of Topology Optimization

Topology optimization (TO) was applied using a density-based Solid Isotropic Material with Penalization (SIMP) formulation to minimize component mass while preserving structural performance. The optimization goal was defined as Best Stiffness-to-Weight Ratio (SolidWorks Simulation Topology StudyPremium 2023), with convergence considered achieved when the relative difference in the objective function between the last two iterations fell below 0.1%. Cylindrical protected zones with a minimum wall thickness of 2 mm were defined at the load application and support interfaces as non-design regions, and symmetry boundary conditions were applied as additional geometric constraints to ensure a symmetric, manufacturable material distribution. The bearing seats, shaft support interfaces, and load application regions were defined as non-design domains to ensure that functional interfaces remained unchanged during material removal. The resulting optimized geometry includes internal cavities. Material is retained primarily near the end interfaces, where loads and constraints are transferred to the structure.

Compared with the baseline configuration, the topology-optimized model demonstrates improved structural efficiency, as a reduced material volume sustains comparable stress levels. The optimized material distribution follows the principal load path, resulting in more uniform stress utilization across the remaining structure, particularly near the load-transfer regions at the end connections.

Despite significant geometric modification, the displacement field maintains a symmetric deformation pattern along the longitudinal axis, confirming that removing low-energy regions does not alter the global deformation mode under axial loading conditions.

### 2.5. Application of Generative Design

Generative design was used to improve the structural performance and material efficiency of the PA6 conveyor roller component. This approach combines topology optimization algorithms with parametric modeling techniques to generate alternative geometric configurations that meet predefined functional requirements [30]. By iteratively redistributing material within the design space, GD enables the development of lightweight structures while maintaining the required strength and stiffness.

The generative design settings applied in this study were as follows: loading conditions and boundary constraints remained constant throughout the optimization process; external forces and their directions were specified as fixed inputs; material properties were held constant; and the algorithm was permitted to modify only the material distribution within the allowable design region, using the topology-optimized geometry as the initial design space.

**Figure 2:**
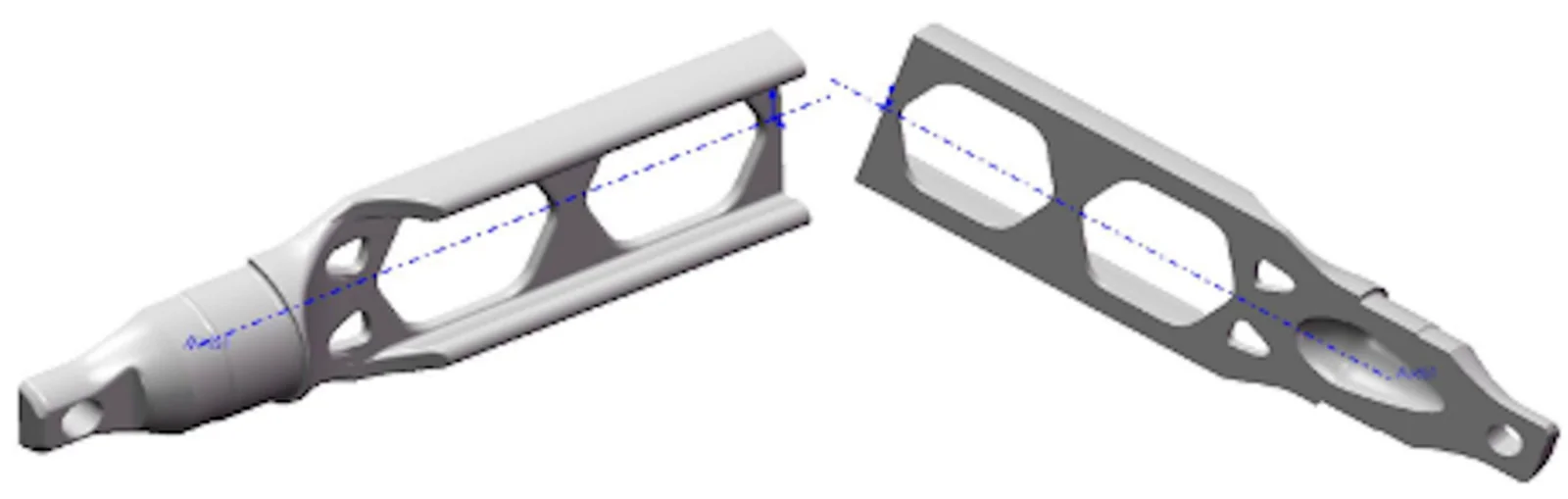
Final geometry of the generative design model (phase C), showing longitudinal and transverse symmetry planes used to define the quarter-symmetry FEA model.

The geometry resulting from the topology optimization stage was used directly as the input geometry for the generative design process, without manual reconstruction or reinterpretation of the topology-optimized shape. The resulting configuration is a material-efficient structure in which material is retained primarily along the principal load paths, while low-utilization regions are removed. The model is symmetric with respect to both longitudinal and transverse planes, consistent with the applied loading and boundary conditions. Reinforced end regions were maintained to ensure reliable load transfer and compatibility with the defined supports. The final optimized geometry is shown in Figure 2. The geometry generated by the generative design process was subsequently refined to achieve a relatively regular, manufacturable form suitable for injection molding, while preserving the load path-based material distribution identified during optimization.

### 2.6. Numerical Analysis

**Figure 3:**
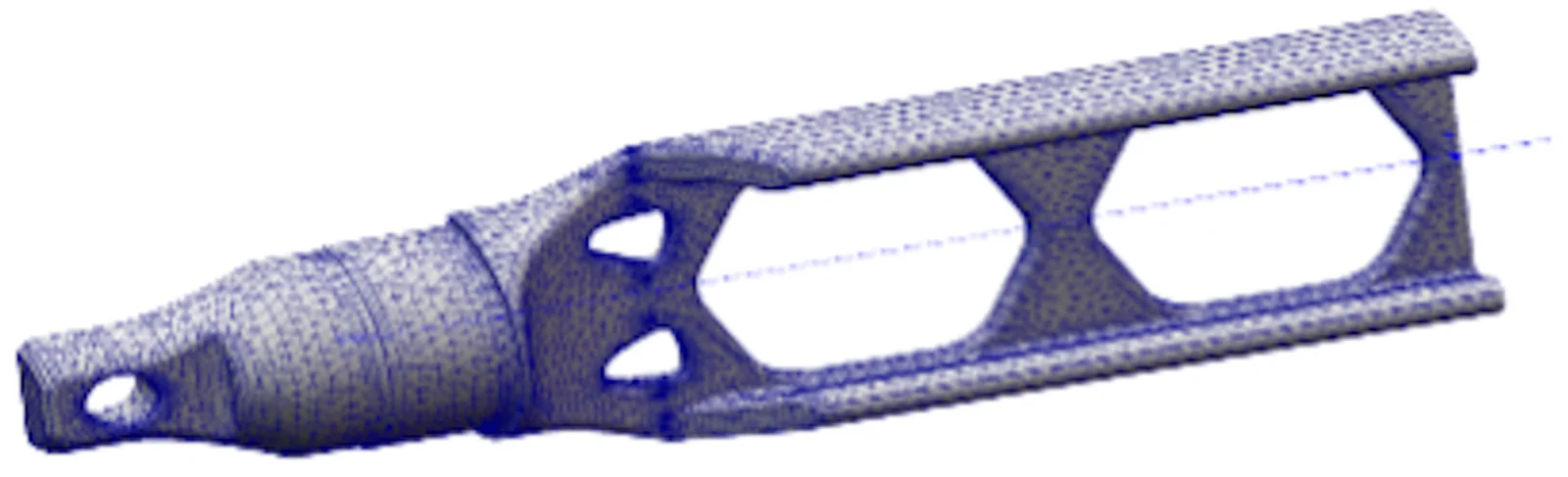
Finite element mesh of the quarter-symmetry model (tetrahedral elements; maximum size 4 mm, minimum size 0.2 mm).

For validating results obtained from topology optimization, the generated models were subjected to numerical analyses using FEA to verify mechanical properties and assess the behavior of components under simulated stresses, deformations, and representative operating conditions by dividing them into smaller, more manageable pieces. Taking advantage of the geometric and loading symmetry to reduce computational cost, every individual element represents a distinct component of the structure, and when combined, these parts closely simulate the overall behavior of the complete structure when subjected to certain loads, limitations, and environmental variables. Due to symmetrical planes in previous results, only one quarter of the geometric model is enough for conducting the work, symmetry boundary conditions are applied on the cut planes to accurate representation of the full model behavior, mesh refinement ensures accurate stress and displacement results, demonstrated in Figure 3.

**Figure 4:**
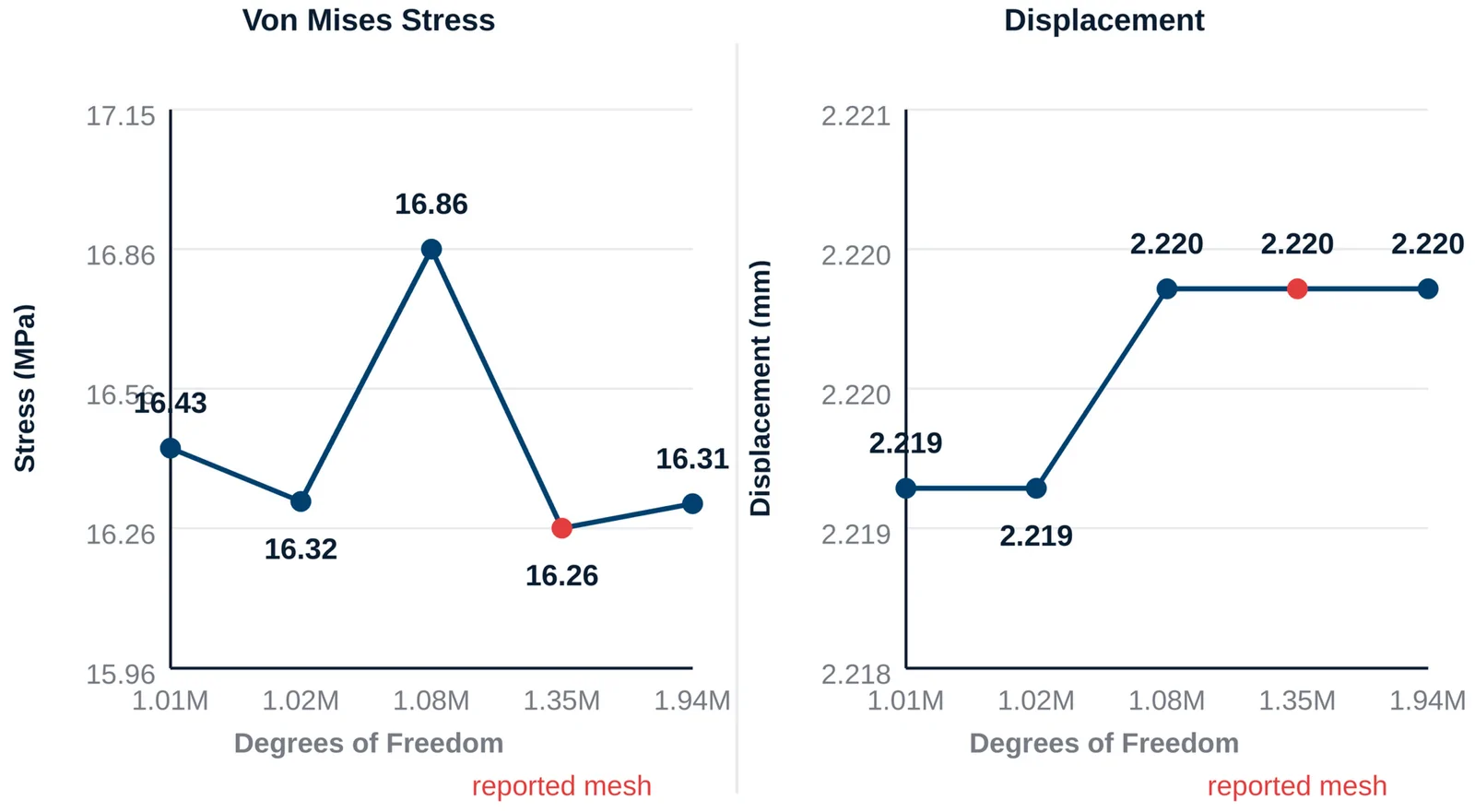
Mesh convergence of (**left**) maximum von Mises stress and (**right**) maximum displacement across five mesh densities. The highlighted point corresponds to the mesh used for all reported results.

The numerical simulations were performed in SolidWorks Simulation (COSMOS-FEA), employing a linear static analysis to determine the stress and displacement fields of the optimized structure. The model was discretized using tetrahedral isoparametric solid elements, with a high-quality mesh defined by 16 Jacobian points per element to ensure accurate representation of curved geometries and stress gradients. A mesh sensitivity study was conducted across five mesh densities, ranging from 1,007,544 to 1,941,612 degrees of freedom, to determine the optimal element size (Table 2, Figure 4). The relative difference in maximum von Mises stress between the selected mesh (1,347,540 degrees of freedom; 4 mm maximum, 0.2 mm minimum element size) and the next finer mesh examined (1,941,612 degrees of freedom) was 0.32%, while the corresponding difference in maximum displacement was below 0.1%, confirming that the selected mesh density provides a mesh-independent solution. Contact between the roller shell, shaft, and bearing interfaces was not explicitly modeled; bearing locations were instead represented as idealized support boundary conditions (Section 2.1), consistent with standard practice for global structural response analysis of this type of component. Since the analysis was performed on a quarter-symmetry model, the resulting mesh consisted of 70,218 elements and 112,295 nodes, corresponding to 336,885 degrees of freedom; when extrapolated to the full geometry, this corresponds to 280,872 elements, 449,180 nodes, and 1,347,540 degrees of freedom. This mesh density was found to provide a satisfactory balance between computational efficiency and solution accuracy, ensuring convergence of the stress and displacement results.

## 3. Results and Discussion

**Figure 5:**
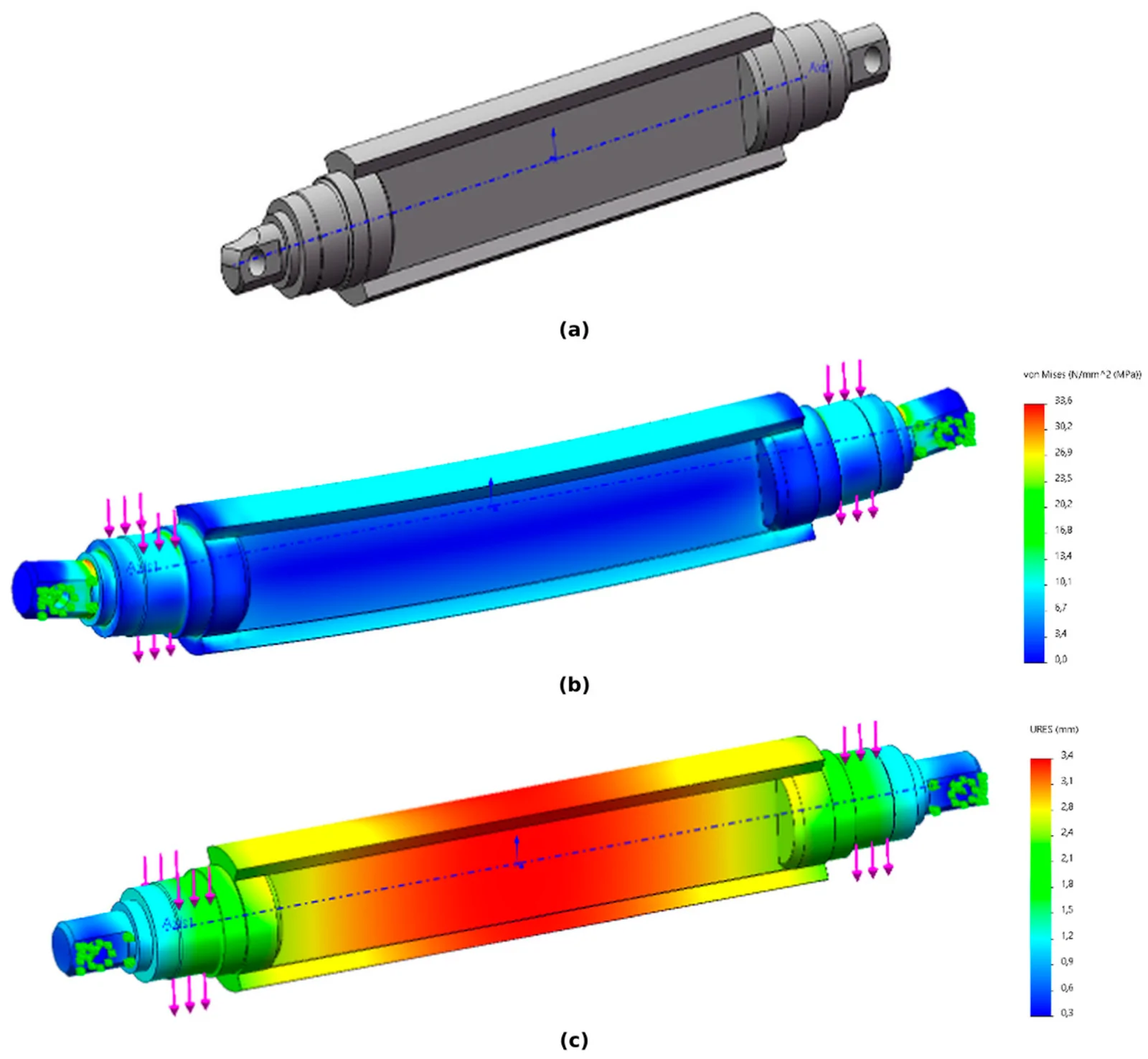
Phase A (conventional baseline model): (**a**) model geometry; (**b**) von Mises stress distribution; (**c**) displacement field. Maximum stress 33.6 MPa; maximum displacement 3.4 mm.

**Figure 6:**
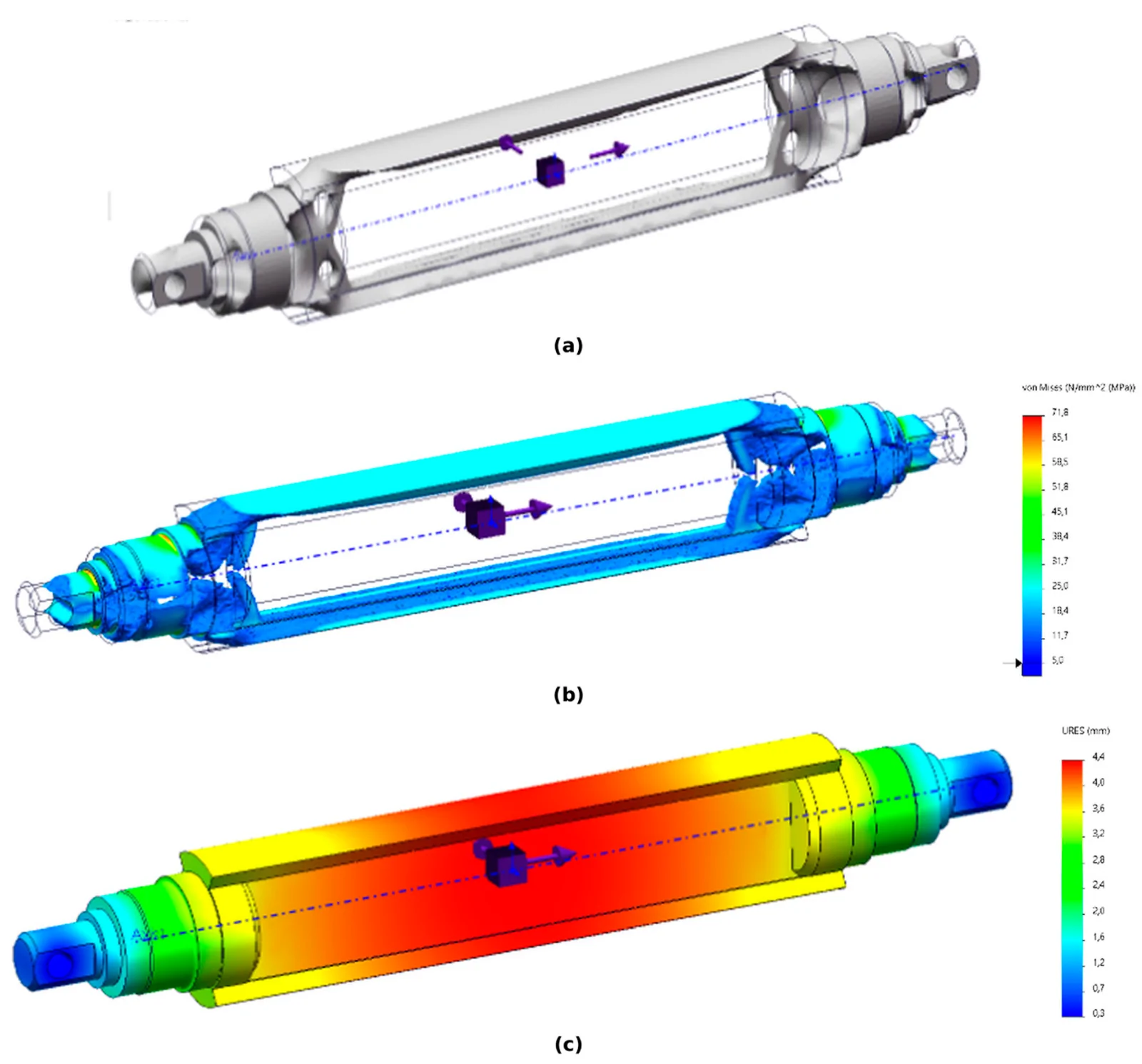
Phase B (topology-optimized model): (**a**) model geometry; (**b**) von Mises stress distribution, displayed above the 5 MPa threshold; (**c**) displacement field. Maximum stress 71.8 MPa; maximum displacement 4.4 mm.

**Figure 7:**
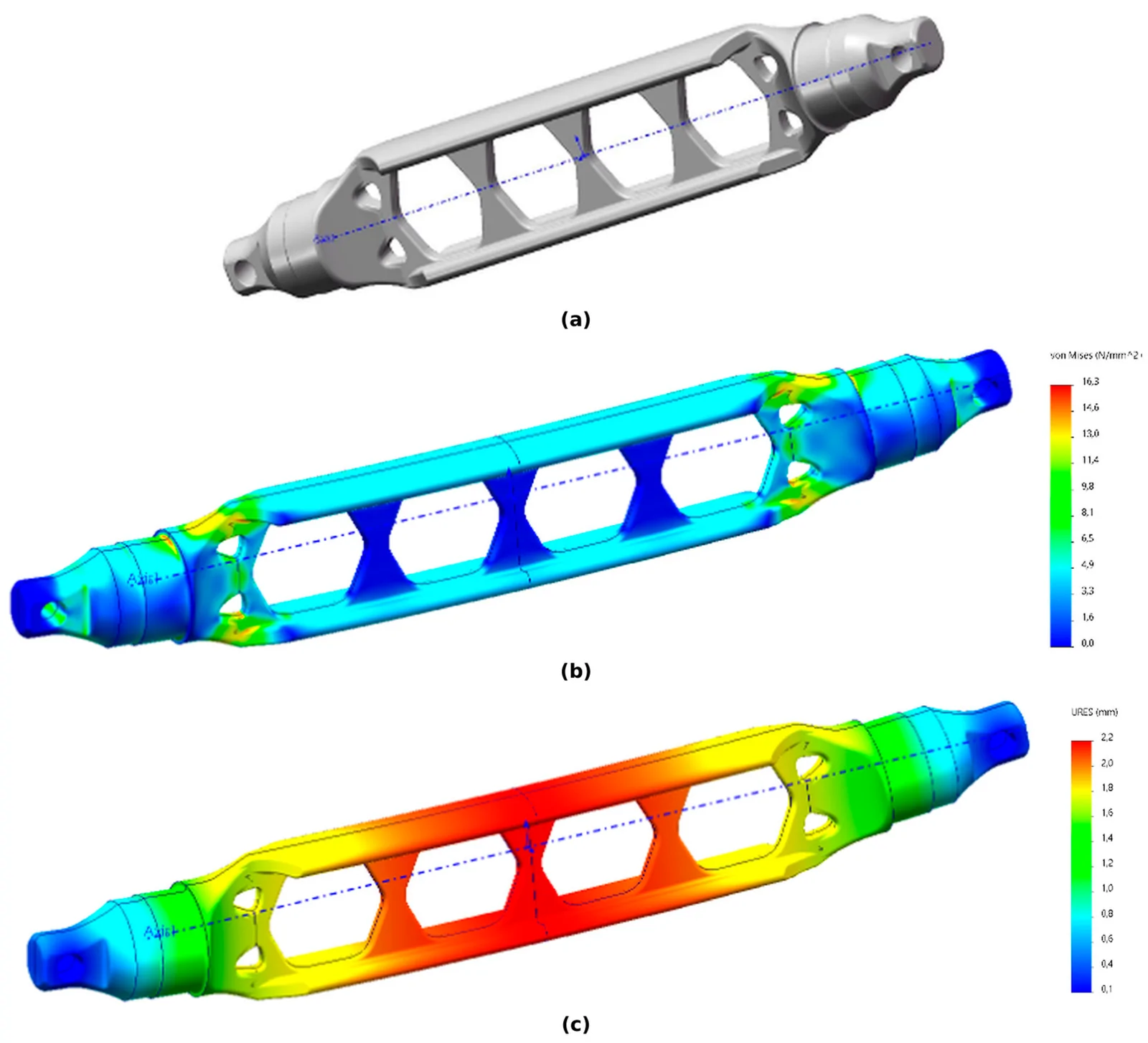
Phase C (generative design model): (**a**) model geometry; (**b**) von Mises stress distribution; (**c**) displacement field. Maximum stress 16.3 MPa; maximum displacement 2.2 mm.

As a result of generative simulation, a design, and stress and displacement distribution, the mass of the formed design was obtained. The designs and stress and displacement states are illustrated in Figure 5, Figure 6 and Figure 7. The simulation results can be sorted in three different phases.

Development phase A results, corresponding to the conventional (baseline) model, are shown in Figure 5. The model weight was 1345 g. The maximum von Mises stress reached approximately 33.6 MPa, localized at the shoulder regions where the diameter transitions occur, while the maximum displacement of 3.4 mm was recorded at the mid-span of the roller, consistent with the expected response of a uniformly loaded cylindrical beam under symmetric boundary conditions. No abnormal stress concentrations, bending, or torsional effects were observed beyond those expected for this loading configuration, indicating a stable and predictable baseline structural response.

Development phase B results, corresponding to the topology-optimized model, are shown in Figure 6. Only stresses above 5 MPa are displayed, isolating the load-bearing material distribution from elements identified for removal, which carry negligible stress and were excluded from the post-processed stress field. The weight was significantly reduced by introducing internal cavities; the resulting central cavity corresponds to a region of minimal structural utilization, where material was removed with negligible effect on load transfer. Peak stress (71.8 MPa) occurs locally at geometric transitions near the end connections, reflecting the reduced cross-section available for load transfer at the bearing interfaces following material removal, rather than a global redistribution of stress. Because this peak stress exceeds the PA6 yield-strength range of 60–65 MPa reported in Table 1, the topology-optimized model was not considered a final acceptable design, but rather an intermediate configuration used to identify low-utilization regions and guide the subsequent generative design stage. The maximum displacement increased to 4.4 mm, compared with 3.4 mm in the baseline model, indicating that mass reduction at this stage was achieved at the expense of overall stiffness, a trade-off subsequently addressed through generative design in Phase C.

Development phase C results, corresponding to the generative design model, are shown in Figure 7. The final weight was 848 g, while preserving the required structural stiffness through balanced load transfer along the principal load paths. Low stress levels dominate the central part of the structure, with a narrower and more controlled stress range compared to phase B. Stress peaks are lower and smoother than in phase B, remaining localized at sharp curvature changes near the reinforced end regions, consistent with the targeted material retention strategy applied during generative design. The maximum stress of 16.3 MPa represents a significant reduction relative to both the conventional model (33.6 MPa, phase A) and the topology-optimized model (71.8 MPa, phase B). The maximum displacement was observed in the central region of the component and decreased gradually toward the end connections, where boundary conditions are applied. The deformation pattern is smooth and symmetric, with a maximum displacement of 2.2 mm, representing a 35% reduction relative to the baseline model (phase A) and a 50% reduction relative to the topology-optimized model (phase B), recovering the stiffness loss observed in phase B. A summary comparison of mass, maximum stress, maximum displacement, and safety factor across all three development stages is provided in Table 3.

The progressive evolution of the stress field across the three development phases reflects the underlying logic of the applied optimization sequence. In the conventional model (phase A), stress is distributed relatively uniformly along the shaft, with mild concentration at the shoulder regions associated with diameter transitions, a typical response for a uniformly loaded cylindrical beam. Topology optimization (phase B) removed material from low-stress, low-strain energy regions identified in the baseline model, which is mechanically consistent with the resulting shift in peak stress: with less cross-section available to transfer load at the bearing interfaces, the maximum von Mises stress increased substantially (71.8 MPa) and became sharply localized at the geometric transitions near the end connections, rather than distributed globally. Generative design (phase C) addressed this concentration directly by selectively retaining material along the principal load paths and at the reinforced end regions, which is reflected in the smoother, lower, and more uniformly distributed stress field of the final model (16.3 MPa maximum). This sequence illustrates that topology optimization and generative design are complementary rather than redundant steps: topology optimization identifies where material can be removed, while generative design determines how the remaining material should be reorganized to restore an efficient load path. This behavior is not imposed explicitly during the design process, but emerges from the interaction between the applied mechanical loading, finite element-based evaluation, and the geometric constraints defined for the generative design stage. Unlike topology optimization, which redistributes material based on a continuous density field driven primarily by a single compliance minimization objective, generative design explores a broader design space by evaluating multiple candidate geometries against combined stiffness, mass, and manufacturability criteria. This allows the algorithm to selectively reinforce load-bearing regions identified as critical during the topology optimization stage, rather than uniformly redistributing the previously removed material.

A central finding of this study is that mass reduction and stiffness did not improve monotonically across the optimization sequence. While topology optimization alone reduced component mass, it simultaneously increased maximum displacement from 3.4 mm (phase A) to 4.4 mm (phase B), indicating a temporary loss of stiffness as a direct consequence of material removal without corresponding structural reinforcement. This behavior highlights a limitation of applying topology optimization in isolation: aggressive mass reduction can compromise stiffness if the resulting geometry is not subsequently refined. Generative design (phase C) reversed this trend, reducing maximum displacement to 2.2 mm, a 35% reduction relative to the baseline model and a 50% reduction relative to the topology-optimized stage, while further reducing mass to 848 g (37% below baseline). This demonstrates that the sequential TO–GD workflow not only preserves but improves stiffness relative to the original design, despite the substantial mass reduction. In terms of structural safety, the maximum von Mises stress of the final model (16.3 MPa) remains well below the material’s yield strength (60–65 MPa), corresponding to a safety factor of approximately 3.7 against yielding under the applied static load. This margin indicates that the optimized component retains a considerable structural reserve beyond the prescribed operating conditions, supporting its suitability for practical implementation. The final geometry remains compatible with conventional injection molding, consistent with standard PA6 production practice, while also being suitable for additive manufacturing as an alternative production route if further design complexity were required.

The mass reduction achieved in this study (37%) is consistent with results reported for similar lightweight optimization efforts on conveyor roller and idler components. Comparable studies on polymer composite roller conveyors using glass fiber-reinforced composites reported mass reductions of up to approximately 42% in the roller component alone [31]. Generative design applied to conveyor support brackets in aluminum alloys achieved substantial volume reduction while maintaining safety factors between approximately 2.2 and 13.4 depending on loading conditions [32], in broad agreement with the safety margin obtained in the present study. Similarly, generative design case studies on other rotating machine elements, such as gear bodies, have reported mass reductions in the range of 37–46% while maintaining acceptable deformation levels [33], closely matching the magnitude of mass reduction obtained here. These comparisons suggest that the optimization outcomes obtained for the PA6 conveyor roller are consistent with the range of performance improvements reported for generative design and topology optimization applied to comparable load-bearing components, despite differences in material class and loading configuration. Notably, a closely related trend has been reported by Barbieri and Muzzupappa [21], who observed a comparable combination of mass reduction (approximately 38%), displacement reduction (approximately 42%), and a resulting factor of safety of 3.5 in case studies applying sequential topology optimization and generative design to metallic components (a titanium rocker and an automotive brake pedal). Although obtained for a different material class and loading configuration, the agreement in the order of magnitude of these metrics with the present polymer-based case study suggests that the observed mass–stiffness trade-off, and its resolution through sequential TO–GD application, may reflect a general characteristic of this optimization workflow rather than a material-specific effect. It should nonetheless be noted that direct numerical comparison of percentage mass reduction across studies employing different base materials, component geometries, and loading regimes should be interpreted with caution, as the achievable mass reduction depends strongly on the structural efficiency of the baseline design rather than on the optimization method alone. The comparisons presented here therefore indicate that the present results fall within a plausible and previously reported range for generative design and topology optimization applied to load-bearing components, rather than establishing direct quantitative equivalence between material classes.

While the individual application of topology optimization and generative design has been established across a range of engineering domains, the present study specifically demonstrates their sequential, FEA-validated application to a polymer, load-bearing rotating conveyor component under representative industrial service loading conditions, a combination not identified, to the authors’ knowledge, in the literature reviewed for this study. Several limitations should be acknowledged. The applied loading condition (2000 N per bearing) represents a static, representative service load consistent with analytical estimates for conveyor idler rollers; cyclic or fatigue loading, which is relevant for rollers operating continuously over extended service periods, was not evaluated in this study and should be addressed in future work. Although fatigue behavior was not evaluated in this study, the substantial safety margin obtained under static loading (SF ≈ 3.7) suggests that the optimized geometry retains structural reserve beyond the prescribed static service conditions. This margin should not, however, be interpreted as a direct indicator of fatigue performance, since polymer fatigue is governed by distinct, cycle-dependent damage mechanisms not captured by a linear static safety factor [34]. This study was conceived as a numerical case study, aimed at establishing and quantifying the sequential topology optimization–generative design methodology under representative service loading; as such, the mechanical properties used for PA6 were obtained from manufacturer datasheets rather than independent experimental characterization of the specific material batch used in this study. While datasheet values are standard practice for preliminary structural design, experimental validation would further strengthen confidence in the numerical predictions. Such validation is planned as a subsequent phase of this research and would involve full-field measurement of a manufactured prototype using Digital Image Correlation under representative static loading, enabling direct, spatially resolved comparison between measured and finite-element-predicted stress and displacement fields. Additionally, the use of a quarter-symmetry model, while computationally efficient and justified by the geometric and loading symmetry of the component, assumes idealized boundary conditions that may not fully capture asymmetric effects such as belt misalignment or uneven load distribution across the roller length. Despite these limitations, the results demonstrate that the combined application of topology optimization and generative design provides an effective and reproducible methodology for reducing the mass of polymer-based conveyor components while maintaining adequate structural performance, with direct implications for reducing energy consumption and material use in belt conveyor systems.

## 4. Conclusions

This study presented a structured numerical methodology for reducing the mass of a PA6-based conveyor idler roller through the sequential application of topology optimization and generative design, validated by finite element analysis. The optimization process aimed to identify the most favorable balance between structural stiffness and component mass under representative service loading conditions.

Starting from a conventional baseline model with a mass of 1345 g, the application of topology optimization followed by generative design reduced the final component mass to 848 g, corresponding to a 37% mass reduction. This mass reduction was achieved while simultaneously improving structural stiffness; maximum displacement decreased from 3.4 mm in the baseline model to 2.2 mm in the final design, a 35% reduction, despite an intermediate increase in displacement to 4.4 mm during the topology optimization stage alone. This result indicates that generative design played a critical corrective role, restoring and ultimately improving stiffness relative to the original design after the temporary stiffness loss introduced by topology optimization. The maximum von Mises stress in the final model (16.3 MPa) remained well below the material’s yield strength (60–65 MPa), corresponding to a safety factor of approximately 3.7 under the applied static load, indicating a substantial structural safety margin. These outcomes are consistent with the range of mass and stiffness improvements reported in the literature for generative design and topology optimization applied to comparable load-bearing and conveyor components.

The results confirm that the combined application of topology optimization and generative design constitutes an effective and reproducible methodology for the lightweight redesign of polymer-based conveyor components, with direct implications for reducing material consumption and energy demand in belt conveyor systems. The findings are considered valid within the defined static loading conditions and the material properties adopted from the manufacturer’s datasheet. The results and conclusions presented in this study are therefore applicable to service conditions involving proper belt alignment and uniform material loading. Under asymmetric loading conditions, such as belt mistracking or uneven material distribution, the internal rib structures resulting from generative design may be particularly sensitive to the resulting eccentric loads, and the reported stress and displacement margins should not be assumed to hold without further evaluation. Future work should therefore address cyclic and fatigue loading relevant to the continuous operational service of conveyor rollers, experimental validation of the optimized component through physical testing, and evaluation of asymmetric loading conditions, such as belt misalignment, which were not captured by the quarter-symmetry model adopted in this study.

## Figures and Tables

**Table 1:** Mechanical Properties of PA6 used in the numerical model, obtained from the manufacturer’s datasheet.

Property	Unit	Value
Density	g/cm^3^	1.14
Modulus of Elasticity	MPa	3300
Tensile Strength	MPa	79
Yield Strength	MPa	60–65
Elongation at Break	%	130
Charpy Impact Strength (unnotched)	kJ/m^2^	No break
Charpy Impact Strength (notched)	kJ/m^2^	7
Ball Indentation Hardness	N/mm^2^	155
Flexural Strength	MPa	100

**Table 2:** Mesh convergence study across five mesh densities. The bolded row corresponds to the mesh density used for all reported results in this study.

Degrees of Freedom	Max. Stress (MPa)	Max. Displacement (mm)	Safety Factor
1,007,544	16.43	2.219	3.67
1,020,216	16.316	2.219	3.69
1,075,788	16.856	2.220	3.60
**1,347,540 (reported)**	**16.259**	**2.220**	**3.71**
1,941,612	16.311	2.220	3.70

**Table 3:** Summary of mass, maximum stress, maximum displacement, and safety factor (relative to a conservative yield strength of 60 MPa) across the three development stages. Percentage changes are calculated relative to the baseline model (Phase A).

Design Stage	Mass (g)	Δ Mass (%)	Max. Stress (MPa)	Max. Displacement (mm)	Δ Displacement (%)	Safety Factor
Phase A—Baseline	1345	—	33.6	3.4	—	1.79
Phase B—Topology Optimization	806	−40.1	71.8	4.4	+29.4%	0.84
Phase C—Generative Design	848	−37.0	16.3	2.2	−35.3%	3.68

## Data Availability

The original contributions presented in this study are included in the article. Further inquiries can be directed to the corresponding author.

## Abbreviations

The following abbreviations are used in this manuscript:

PA6	polyamide-6
CAD	Computer-Aided Design
CAM	Computer-Aided Manufacturing
GD	Generative design
FEA	Finite Element Analysis
TO	Topology optimization
SIMP	Solid Isotropic Material with Penalization
